# Insight into crRNA Processing in *Streptococcus mutans* P42S and Application of SmutCas9 in Genome Editing

**DOI:** 10.3390/ijms26052005

**Published:** 2025-02-25

**Authors:** Cas Mosterd, Sylvain Moineau

**Affiliations:** Département de Biochimie, de Microbiologie et de Bio-Informatique, Faculté des Sciences et de Génie, Université Laval, Quebec City, QC G1V 0A6, Canada

**Keywords:** CRISPR-Cas9, PAM, genome editing, *Streptococcus mutans*, *Lactococcus cremoris*, bacteriophage

## Abstract

CRISPR-Cas is an adaptive immune system found in bacteria and archaea that provides resistance against invading nucleic acids. Elements of this natural system have been harnessed to develop several genome editing tools, including CRISPR-Cas9. This technology relies on the ability of the nuclease Cas9 to cut DNA at specific locations directed by a guide RNA. In addition, the nuclease activity of Cas9 requires the presence of a short nucleotide motif (5′-NGG-3′ for Cas9 from *Streptococcus pyogenes*) called PAM, flanking the targeted region. As the reliance on this PAM is typically strict, diverse Cas9 variants recognising different PAM motifs have been studied to target a broader range of genomic sites. In this study, we assessed the potential of Cas9 from *Streptococcus mutans* strain P42S (SmutCas9) in gene editing. SmutCas9 recognises the rarely targeted 5′-NAA-3′ and 5′-NGAA-3′ PAMs. To test its efficacy, two genes of the virulent lactococcal phage p2 were edited, thereby demonstrating the potential of SmutCas9 for gene editing purposes, particularly in AT-rich genomes. Sequencing of total RNA also revealed the RNA components of this system, allowing further molecular characterisation of the type II-A CRISPR-Cas system of *S. mutans*.

## 1. Introduction

CRISPR-Cas is a natural antiviral defence mechanism found in approximately 45% of all bacteria [1]. CRISPR (Clustered Regularly Interspaced Short Palindromic Repeats) consists of an array of palindromic nucleotide repeats, each separated by variable sequences called spacers. These spacers often share homology with viral and plasmid sequences [2,3,4]. CRISPR arrays are typically flanked by *cas* (CRISPR-associated) genes [5,6]. Together, CRISPRs and *cas* genes provide resistance against mobile genetic elements such as phages [7,8], by precisely cleaving invading nucleic acids [9]. The CRISPR arrays are transcribed and processed into short crRNAs, which guide Cas nucleases to their target via base pairing [10]. CRISPR-Cas systems have been extensively studied and classified in various classes, types, and subtypes. Class 1 CRISPR-Cas systems contain multiple Cas proteins that form an effecter complex to cleave nucleic acids, whereas Class 2 systems rely on a single Cas effector protein [1].

For over a decade, a significant portion of CRISPR-Cas research has focused on its potential applications in biotechnology, particularly in genome editing. While Class 1 systems have been explored for genome editing purposes [11,12,13,14,15], Class 2 systems have been more widely adopted due to their simpler gene architecture. Among Class 2 systems, the most studied for genome editing are type II systems, featuring Cas9 as the effector protein [16], and type V, with Cas12a (formerly known as Cpf1) as the effector protein [17,18]. Both Cas9 and Cas12a can be programmed to cleave specific DNA sequences when guided by a crRNA that directs the nuclease to its target. These proteins have proven to be highly versatile tools for genome editing [19]. The most used has been Cas9 from *Streptococcus pyogenes* (SpyCas9) and numerous Cas9-mediated functions and implementation strategies have been investigated [20,21]. However, the range of targetable sequences is often constrained by the requirement for protospacer adjacent motifs (PAMs) [21,22,23].

In genome editing, the PAM requirement enhances CRISPR targeting precision by preventing DNA cutting in the absence of a perfect PAM sequence flanking the targeted site. However, this Cas9-dependent PAM specificity can also be a limitation, as it restricts the number of accessible targets within a given DNA region. To overcome this limitation and expand the range of targetable sequences, various natural Cas9 proteins with different PAM requirements have been studied to assess their potential in genome editing [24,25,26,27]. Additionally, Cas9 and Cas12a variants have been engineered to modify or relax their PAM requirements [28,29,30,31,32,33,34,35,36]. However, reducing PAM specificities decreases the precision of the system, thus increasing the likelihood of off-target effects, a concern in genome editing technology [37,38].

The Cas9 nuclease from *Streptococcus mutans* strain P42S (SmutCas9) has been previously shown to recognise the PAM sequences 5′-NAA-3′ and 5′-NGAA-3′ [39,40]. The type II-A CRISPR-Cas system in this *S. mutans* strain has also been demonstrated to interfere with plasmid transformation and contribute to antiviral defence [39,40]. The CRISPR-Cas system of *S. mutans* strain UA159 has been utilised for genome editing through self-targeting CRISPR spacers [41]. However, this Cas9 variant recognises the PAM sequence 5′-NGG-3′, the same as the widely used SpyCas9.

The CRISPR-SpyCas9 technology has previously been applied in *Lactococcus cremoris* [42], a bacterial species known for its diverse antiviral defence mechanisms but typically lacking CRISPR-Cas systems [43,44], to knock out non-essential genes in the genome of a virulent lactococcal siphophage. Phage p2, a member of the *Skunavirus* genus [45,46,47], infects *L. cremoris* MG1363, a model strain for studying low-GC gram-positive bacteria [48,49]. In this study, we assess the potential of SmutCas9 for CRISPR-Cas9 genome editing by targeting two genes of the lactococcal phage p2 that encode proteins of unknown function.

## 2. Results

The activity and PAM preferences of SmutCas9 were previously established, but information about the crRNA and tracrRNA sequences required for its functionality remained unknown [39].

### 2.1. RNA Sequencing

In order to obtain the crRNA and tracrRNA sequences, total RNA was extracted from *S. mutans* P42S and sequenced, yielding 9,374,170 reads. The native CRISPR array of *S. mutans* P42S consists of six repeats and five spacers. Notably, the last repeat (at the 3′-end) differs by a single nucleotide (C > T). Reads corresponding to four of the five expected crRNAs were detected. Specifically, crRNAs were identified for spacers 1, 2, 3, and 5, starting from the 5′-end of the CRISPR locus. The absence of reads for spacer 4 remains unexplained. The crRNAs corresponding to spacers 1 (crRNA1), 2 (crRNA2), and 3 (crRNA3) were 43 nucleotides long, comprising the last 19 nucleotides of the spacer followed by the first 24 nucleotides of the repeat. The crRNA of spacer 5 (crRNA5) was 39 nucleotides long, including the last 19 nucleotides of the spacer but followed by only the first 20 nucleotides of the repeat.

RNA sequencing also identified the first 63 nucleotides of the tracrRNA. Previous studies determined that the tracrRNA in *S. mutans* UA159 was 107 nucleotides long [50,51], while it was estimated to be 93 nucleotides in *S. mutans* P42S [39]. This suggests that the detected 63-nucleotide tracrRNA may have either been processed or incomplete. Subsequent poly-A tailing of RNA, followed by cDNA library construction, PCR with a poly-T primer, and subsequent sequencing, revealed that the tracrRNA in *S. mutans* P42S was 88 or 89 nucleotides long. The sequences of the crRNAs and tracrRNA are presented in Table 1.

### 2.2. Gene Editing of Phage p2

To evaluate the potential of SmutCas9 in genome editing, plasmid pTRKL2-SmutCas9 was constructed as described in the Materials and Methods section. This construct consists of the *tracrRNA*, *cas9,* and a repeat-spacer-unit (R-S-R) from *S. mutans* P42S along with the pTRKL2 backbone. The full sequence of the plasmid was confirmed using Illumina sequencing. Once pTRKL2-SmutCas9 was obtained, new constructs were generated by replacing the original non-targeting spacer within the R-S-R (repeat-spacer-repeat) with a spacer designed to target a protospacer (flanked by the appropriate PAM) in either *orf49* or *orf44* of lactococcal phage p2. The R-S-R regions of the resulting pTRKL2-SmutCas9-49 and pTRKL2-SmutCas9-44 constructs were amplified by PCR and confirmed by sequencing.

#### 2.2.1. Editing *orf49* of Phage p2

The plasmids pTRKL2-SmutCas9 and pTRKL2-SmutCas9-49 were transformed into *L. cremoris* MG1363-pKO49, a strain carrying a repair template with a truncated version of *orf49*, designed for homologous recombination with the infecting and targeted phage p2. In addition, the plasmids pL2Cas9 (SpyCas9), pL2Cas9-49 (SpyCas9 with a spacer targeting *orf49*) [42], and pTRKL2 (empty vector) were transformed into *L. cremoris* MG1363-pKO49 as controls. All strains were confirmed through plasmid analysis.

**Figure 1:**
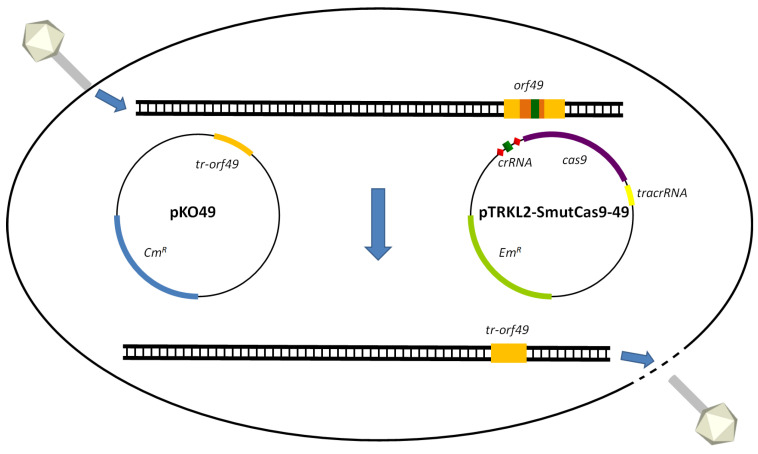
Editing of *orf49* of the lactococcal phage p2 using SmutCas9. At the top of the figure, the injection of phage p2 genome into *L. cremoris* MG1363-pKO49-SmutCas9-49 is illustrated. *orf49* of phage p2 is highlighted in yellow in the linear genome. The part of the gene to be deleted is in orange and the protospacer is in green. Plasmid pTRKL2-SmutCas9-49 contains a spacer (green) that targets the protospacer present in *orf49*. SmutCas9 will cut the phage gene at this position. The plasmid pKO49 contains a truncated version of *orf49* (*tr-orf49*). *orf49* will be exchanged for *tr-orf49* via homologous recombination between pKO49 and the phage p2 genome. The edited phage will then lyse the cell as this phage gene is non-essential for replication in *L. cremoris* MG1363 under laboratory conditions and the targeting spacer is no longer recognising the mutated phage genome.

To edit *orf49* from phage p2 (schema of the protocol is presented in Figure 1), the five *L. cremoris* MG1363-pKO49 derivatives, each transformed with one of the five pTRKL2 constructs, were infected with p2 using a double-layer plaque assay. The efficiency of plaquing (EOP) was calculated by dividing the phage titres obtained on strains carrying the p2-specific spacer (SmutCas9-49 and pL2Cas9-49) by the titres on strains harbouring their non-specific counterparts (SmutCas9 and pL2Cas9). This resulted in an EOP value of 0.85 for SmutCas9-49/SmutCas9 and 0.05 for pL2Cas9-49/pL2Cas9.

The *orf49* region of 100 phage p2 plaques, obtained from each of the five *L. cremoris* MG1363-pKO49 strains harbouring pTRKL2 constructs, was analysed by PCR. Samples were loaded onto a gel, and based on the observed band patterns, each sample was categorised as wild-type (WT, band of 1803 bp), deletion mutant (band of 1671 bp), or a mixed population containing both WT and deletion mutants (bands of 1803 bp and 1671 bp). The summary of the results is presented in Table 2. 

For the strain carrying pL2Cas9-49, all plaques contained the truncated version of *orf49*, confirming the efficacy of SpyCas9 in *Lactococcus*. For *L. cremoris* MG1363 transformed with pTRKL2-SmutCas9-49, all plaques contained a mixture of the wild-type *orf49* and its truncated version, revealing that SmutCas9 is also functional in *L. cremoris* but less efficient than SpyCas9. To obtain pure deletion mutants, plaques from strain pTRKL2-SmutCas9-49 were re-suspended in buffer and used to re-infect the same strain. The resulting lysate was subjected to another round of plaque assay, readily yielding plaques containing either phage p2 or the pure *orf49* deletion mutant (p2Δ49).

#### 2.2.2. Editing *orf44* of Phage p2

Similar to the approach used for *orf49*, the plasmids pTRKL2-SmutCas9 and pTRKL2-SmutCas9-44 were transformed into *L. cremoris* MG1363-pKO44. pL2Cas9-44, pL2Cas9, and pTRKL2 were also transformed into *L. cremoris* MG1363-pKO44 in parallel as controls. The five derivatives of *L. cremoris* MG1363-pKO44 were infected with phage p2 using double-layer plaque assays, and the results are summarised in Table 3. The EOP values were 0.96 for SmutCas9-44/SmutCas9 and 1.2 × 10^−4^ for pL2Cas9-44/pL2Cas9.

Once again, *orf44* from 100 phage p2 plaques, obtained from each of the five *L. cremoris* MG1363-pKO44 strains carrying pTRKL2 constructs, was analysed by PCR. The samples were then loaded onto a gel and classified as WT (905 bp band), deletion mutant (705 bp band), or mixed population (both bands). Surprisingly, with the exception of one plaque from pL2Cas9-44 that contained the wild-type *orf44*, all tested plaques tested showed a mixture of the wild-type and truncated version *orf44*. Similar to p2Δ49, a pure deletion mutant phage, p2Δ44, was obtained by re-suspending phage plaques from pTRKL2-SmutCas9-44 and re-infecting the pTRKL2-SmutCas9-44 strain.

## 3. Discussion

The biological activity of the type II-A CRISPR-Cas system of *S. mutans* P42S was previously investigated [39,40]. Among the findings, it was shown that the system can interfere with plasmid transformation and that SmutCas9 recognises the PAM sequences 5′-NAA-3′ and 5′-NGAA-3′. In this study, we further explored the molecular details of this CRISPR-Cas system and assessed the potential of SmutCas9 for genome editing.

The RNA sequences of the type II-A CRISPR-Cas system in *S. mutans* P42S revealed the presence of four out of the five expected crRNAs in the whole-cell RNA extracts. Three of these crRNAs were 43 nucleotides in length while the fourth, corresponding to the last spacer in the native CRISPR array, was 39 nucleotides long. Previous studies identified the processed crRNAs of *S. mutans* UA159 as being 42 nucleotides [50,51].

The tracrRNA was found to be either 88 or 89 nucleotides long, which is 4 to 5 nucleotides shorter than previously estimated based on sequence analysis of the putative full length, unprocessed tracrRNA. This discrepancy arose because the first four nucleotides of the predicted tracrRNA were missing from the RNA sequencing reads [39]. To determine the 3′-end of the tracRNA, a poly-A tail was added to the RNA using poly(A) polymerase. The predicted full tracrRNA had an adenine as the final nucleotide, making it unclear whether the adenine at position 89 was the final nucleotide of the tracrRNA or the first nucleotide of the poly-A tail.

**Figure 2:**
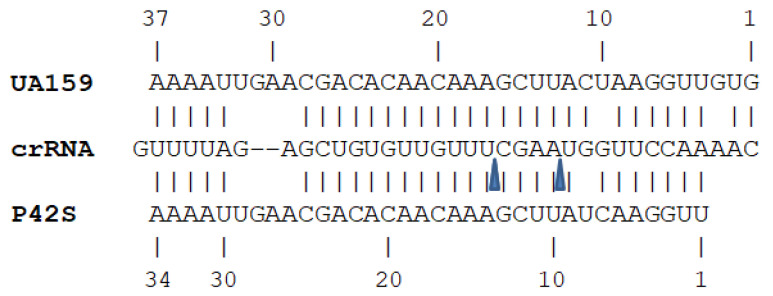
Complementarity between crRNA and tracrRNA of *S. mutans* P42S and UA159. Predicted processing sites of crRNAs are highlighted by arrowheads.

In *S. mutans* UA159, the tracrRNA was previously reported to range from 80 nucleotides (processed) to 102 nucleotides (non-processed) [50,51]. The unprocessed tracrRNA of *S. mutans* P42S is shorter than that of UA159 due to two small deletions and the absence of the first four nucleotides, which were undetected in RNA sequencing. Compared to the tracrRNA of P42S, the tracrRNA of *S. mutans* UA159 has slightly greater complementarity to the repeat regions of its crRNA. However, the tracRNA of P42S still exhibits a critical 25-nucleotide region with 24 nucleotides of complementarity to the crRNA repeat region, which is essential for directing crRNA maturation [51]. The complementarity is illustrated in Figure 2. In addition, based on the 3′ ends of the crRNAs, the predicted crRNA processing sites are highlighted in the same figure.

The plasmid pTRKL2-SmutCas9 was designed to assess the potential of Cas9 from *S. mutans* P42S for viral gene editing. The first indicator of its effectiveness was the level of phage resistance it conferred, measured by EOPs. Based on the EOP values, pTRKL2-SmutCas9 did not seem to contribute to phage resistance.

Still, we investigated whether pTRKL2-SmutCas9 could facilitate the selection of phage mutants. To explore this, we analysed *orf49* of phage p2 by examining plaques from infected *L. cremoris* MG1363-pKO49 strains by PCR. Remarkably, all 100 plaques that infected the pTRKL2-SmutCas9-49 strain contained a mixed population of both the wild-type phage p2 and mutant phage p2Δ49. The phage p2Δ49 was easily isolated after one subsequent round of phage purification. Interestingly, phage p2Δ49 was also recovered at low levels from the control strain pTRKL2-SmutCas9, even though the spacer in this construct did not target the phage. This likely occurred due to homologous recombination between the incoming phage genome and the repair template, even in the absence of Cas9 or a targeting spacer. Nevertheless, the presence of the spacer significantly increases the number of phage mutants (100% vs. 6%), confirming the activity of SmutCas9.

As expected from a previous study, pL2Cas9-49 (SpyCas9) facilitated the rapid isolation of p2Δ49, with all 100 PCR-analysed plaques containing a dominant population of p2Δ49. Interestingly, as observed with pTRKL2-SmutCas9, a small percentage (2%) of p2Δ49 was recovered in the control pL2Cas9, despite the absence of selective pressure against *orf49*. Similarly, p2Δ49 was detected (4%) in the control pTRKL2, even though it encoded neither SmutCas9 nor SpyCas9.

*orf44* was the next target for gene editing. In a previous study, a mixed population of wild-type phage p2 and p2Δ44 was observed using pL2Cas9-44 (SpyCas9) [52]. Surprisingly, this phenotype was not only present in all 100 tested plaques of pTRKL2-SmutCas9-44 and nearly all tested plaques (99/100) of pL2Cas9-44, but also in all 100 tested plaques of pTRKL2-SmutCas9, pL2Cas9, and pTRKL2. This suggests that all these events are likely the result of recombination rather than the activity of pTRKL2-SmutCas9 or pL2Cas9. Viruses evolve rapidly [53,54], and in some cases, the presence of the repair template alone appears to be sufficient for recombination to occur in phage p2. However, the frequency of recombination events varied significantly between *orf44* and *orf49*. Recombination hotspots are known to exist in viral genomes, and such events can occur in these regions even without DNA damage caused by Cas9 [55].

Overall, both pTRKL2-SmutCas9 and pL2Cas9 could be used for viral gene editing in *Lactococcus*. These two tools employ different Cas9 proteins, which recognise distinct PAM sequences. The SpyCas9 used in pL2Cas9 recognises the 5′-NGG-3′ PAM, which appears 1655 times in the p2 genome. In contrast, SmutCas9 recognises 5′-NAA-3’ (found 6823 times in p2) and 5’-NGAA-3’ (found 1296 times in p2). The 27,595 bp genome of p2 has an AT content of 65.3%, which explains the higher number of target sites for pTRKL2-SmutCas9 compared to pL2Cas9. Of note, recent findings have identified at least four distinct groups of *cas9* genes in *S. mutans*, with two of these groups containing PAM-interacting domains different from those currently known [56]. This suggests that the diversity of PAM sequences recognised by type II-A systems in *S. mutans* is likely greater, potentially revealing new candidates for genome editing in this species.

Current genome editing technology relies heavily on SpyCas9, limiting the range of targetable PAM sequences [57]. Expanding the scope of genome editing, particularly in AT-rich sequences such as the human genome [58], could be achieved by using Cas9 proteins that recognise AT-rich PAMs. In this study, we showed that SmutCas9 can be used to edit viral genes as a proof of concept. Its ability to edit bacterial and eukaryotic genes remains to be explored. The latter is particularly of importance considering the first human-based CRISPR therapy was recently approved [59].

## 4. Materials and Methods

### 4.1. Bacterial Strains, Phages, and Growth Conditions

*S. mutans* P42S, as well as *L. cremoris* MG1363 and its derivatives, were obtained from the Félix d’Hérelle Reference Center for Bacterial Viruses (www.phage.ulaval.ca). *S. mutans* P42S was grown in Brain Heart Infusion (BHI) medium at 37 °C with 5% CO_2_. For growth on solid media, 1.25% agar was added to BHI. *L. cremoris* MG1363-pKO49 and *L. cremoris* MG1363-pKO44 are derivatives of *L. cremoris* MG1363 that carry the plasmids pKO49 or pKO44, which serve as repair templates for phage p2 genes *orf49* or *orf44*, respectively [42,52]. *L. cremoris* strains were grown in M17 medium supplemented with 0.5% glucose (GM17). To maintain pKO44 and pKO49, 5 µg/mL of chloramphenicol was added to the medium. For strains transformed with pTRKL2, pTRKL2-SmutCas9, pL2Cas9, or derivatives, 5 µg/mL of erythromycin was used.

Lactococcal phage p2 was amplified using an exponentially growing culture of *L. cremoris* MG1363. During amplification, the GM17 medium was supplemented with 10 mM CaCl_2_. Phage-infected cultures were incubated at 30 °C until lysis. The resulting lysate was filtered (0.45 μm) and stored at 4 °C until further use. Phage titration was performed using a double-layer plaque assay on solid GM17 medium containing 1.25% agar, with a top agar layer containing 0.75% agar. Both layers were supplemented with 10 mM CaCl_2_.

### 4.2. RNA Extraction and Sequencing

All equipment, reagents, and conditions used were kept RNase-free whenever possible. *S. mutans* P42S was grown until it reached an OD_600_ of 0.6. RNA was extracted from 1 mL aliquots of this culture. The aliquots were centrifuged at maximum speed for 1 min in a tabletop centrifuge, and the supernatant was removed. The resulting pellets were treated with 50 µL of a 60 mg/mL lysozyme solution containing 20% sucrose. Following this, the pellets were re-suspended in 1 mL of TRIzol and incubated at room temperature for 5 min. Next, 200 µL of chloroform was added, and the samples were mixed, incubated at room temperature for 2 min, followed by centrifugation at 12,000× *g* for 15 min and 4 °C. The upper phases were transferred to clean Eppendorf vials and mixed with 500 µL of cold iso-propanol. After incubating at room temperature for 10 min, the samples were centrifuged again at 12,000× *g* for 10 min and 4 °C. The pellets were washed three times with 75% ethanol, air-dried and then re-suspended in water. To remove DNA contamination, the samples were treated with TURBO DNase (Thermo Fisher Scientific, Waltham, MA, USA) following the manufacturer instructions, with the addition of an RNase inhibitor. The RNA samples were further purified using the RNeasy MinElute Cleanup Kit (Qiagen, Hilden, Germany). The purified RNA samples were used to prepare a cDNA library, which was sequenced by Vertis Biotechnology AG (Freising, Germany) on an Illumina (San Diego, CA, USA) NextSeq 500 system using a read length of 75 bp.

To obtain the full tracrRNA sequence, RNA samples were treated with *E. coli* poly(A) polymerase (New England Biolabs, Ipswich, MA, USA), adding a poly-A tail to the 3′-end of the RNA. A cDNA library was prepared using SuperScript III reverse transcriptase (Thermo Fisher Scientific) while replacing the random primers with a poly-T primer. Next, a PCR was performed using the primers tracrRNA_F and PolyT_R (sequences provided in Table 4) and the resulting PCR product was sequenced at the Plateforme de séquençage et de génotypage des génomes of the Centre de recherche du CHUL (Québec, QC, Canada).

### 4.3. Construction of pTRKL-SmutCas9

All oligonucleotides used for cloning are listed in Table 4. The plasmid pUC57-SmutCas9 was synthesised by BioBasic Inc (Markham, ON, Canada). This plasmid contains *tracrRNA*, *cas9*, and a single repeat-spacer-repeat unit of *S. mutans* P42S, cloned between the EcoRV and SmaI restriction sites of pUC57. A 235 bp region upstream of the CRISPR locus in *S. mutans* P42S was included to enable crRNA expression from its natural promoter. The resulting plasmid was 7478 bp in size, with a 4790 bp insert that carries *tracrRNA*, *smutcas9*, and the repeat-spacer-repeat unit. This insert was then cloned into the low copy vector pTRKL2 (6478 bp), which encodes an erythromycin resistance gene and is transformable in both *E. coli* and *L. cremoris* [60].

Both pTRKL2 and SmutCas9 were PCR-amplified with 30 bp overhangs (pTRKL2) and 15 bp overhangs (SmutCas9) to facilitate fusion through Gibson assembly [61]. The primers used for pTRKL2 were pTRKL2_F and pTRKL2_R, while the primers SmutCas9_F and SmutCas9_R were used for SmutCas9. PCR products were separated on agarose gels, and bands of expected size (6384 bp for pTRKL2 and 4814 bp for SmutCas9) were excised and purified using the QIAquick Gel Extraction kit (Qiagen). The two fragments were then assembled using Gibson assembly (New England Biolabs) at a 1:1 molar ratio with a total of 200 ng of DNA. The assembly product was transformed into competent *E. coli* NEB-5α (New England Biolabs) and plated on solid BHI medium containing 150 µg/mL erythromycin. Successful transformants were grown in liquid BHI supplemented with 150 µg/mL erythromycin, and pTRKL2-SmutCas9 was extracted from overnight cultures using the QIAprep Spin Miniprep Kit (Qiagen). The final construct, pTRKL2-SmutCas9, was 11,110 bp in size (full sequence of the plasmid available in Table S1).

To replace the existing spacer in pTRKL2-SmutCas9 with one targeting *orf49* or *orf44* of phage p2, we took advantage of the two BsaI restriction sites within the existing spacer sequence, resulting in cuts in both repeat sequences flanking the spacer. pTRKL2-SmutCas9 was digested with BsaI and dephosphorylated at its 5′ ends using Antarctic Phosphatase (New England Biolabs). The resulting DNA was then precipitated by adding 1/10 volume of 3 M sodium acetate (pH 5.2) and two volumes of 100% ethanol, followed by incubation on ice for 15 min. After centrifugation at 16,000 × *g* for 15 min, the pellet was washed with 70% ethanol, dried, and re-suspended in sterile distilled water.

In parallel, spacers targeting *orf49* and *orf44* were prepared. A total of 100 pmole of each primer (spacer49_F and spacer49_R for *orf49,* and primers spacer44_F and spacer44_R for *orf44*) were phosphorylated using T4 Polynucleotide Kinase (Thermo Fisher Scientific). Annealing was performed by incubating the samples at 95 °C for 5 min, followed by a gradual temperature decrease at the rate of −0.1 °C/s until 25 °C was reached. The samples were then incubated at 25 °C for an additional 10 min [62].

**Figure 3:**
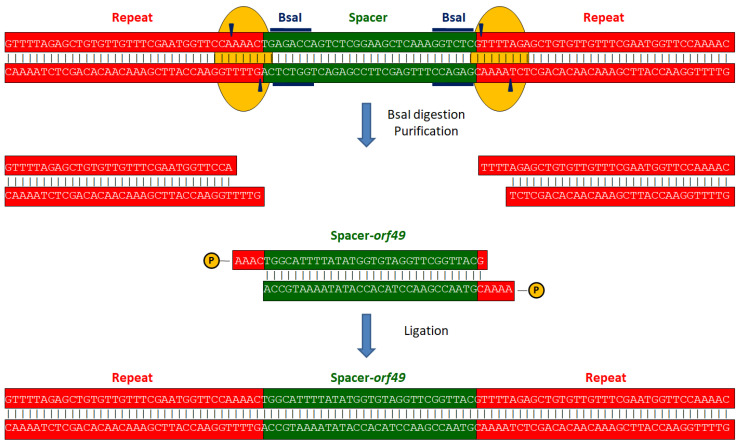
Exchanging the spacer of pTRKL2-SmutCas9. At the top of the figure, the repeat-spacer-repeat unit is shown as found in pTRKL2-SmutCas9. To exchange the spacer for one that targets *orf49*, the first step is the removal of the existing spacer. In blue, the 6 bp restriction sites of BsaI are indicated. The blue arrowheads indicate the exact positions where BsaI (yellow) cuts within the repeat. After purification, a linearised plasmid without the spacer was obtained. Spacer-*orf49* was annealed, phosphorylated, and ligated into linearised pTRKL2-SmutCas9 to obtain pTRKL2-SmutCas9-49.

The digested plasmid and spacer were ligated overnight at 16 °C with a final DNA quantity of 1 µg. The ligation product was transformed into competent *E. coli* NEB-5α. The resulting pTRKL2-SmutCas9-49 and pTRKL2-SmutCas9-44 were extracted from overnight cultures using the QIAprep Spin Miniprep Kit. A visual representation of this process is provided in Figure 3.

As controls, we also included pL2Cas9, pL2Cas9-49 [42], and pL2Cas9-44 [52]. pL2Cas9 is a pTRKL2-based construct carrying the *tracrRNA*, *cas9*, and a repeat-spacer-repeat unit from *S. pyogenes*. pL2Cas9-49 and pL2Cas9-44 are derivative constructs specifically targeting *orf49* and *orf44,* respectively.

### 4.4. Gene Editing of Phage p2

The plasmids mentioned above were transformed into electrocompetent *L. cremoris* MG1363-pKO49 (either pTRKL2, pTRKL2-SmutCas9, pTRKL2-SmutCas9-49, pL2Cas9, or pL2Cas9-49) and *L. cremoris* MG1363-pKO44 (pTRKL2, pTRKL2-SmutCas9, pTRKL2-SmutCas9-44, pL2Cas9, or pL2Cas9-44) as described by Holo and Nes [63]. The transformed strains were then infected with phage p2 and plaques were obtained using a double-layer plaque assay. The resulting plaques were examined by PCR amplification of *orf49* and *orf44*. For *orf49* amplification, primers CB13.42 and p2.27 were used, while primers CB13.10 and CB14.6 were used for *orf44*. Primer CB13.42 anneals within *orf46* of phage p2, whereas primer p2.27 anneals to the non-coding region downstream of *orf49*. Since both primers anneal to the p2 genome, but not to the repair template pKO49, the expected PCR product sizes were 1803 bp for the wild-type *orf49* and 1671 bp for the truncated version. Similarly, primer CB13.10 anneals within *orf43*, and CB14.6 binds to the non-coding region downstream of *orf44*. These primers do not anneal to the repair template pKO44, resulting in a PCR product of 905 bp for the wild-type *orf44* and 705 bp for the truncated version. An overview of the gene editing protocol is shown in Figure 1. The EOP values were calculated by dividing the titres obtained for strains carrying the p2-specific spacer (SmutCas9-49, pL2Cas9-49, SmutCas9-44, and pL2Cas9-44) by the titres of strains harbouring their non-specific counterparts (SmutCas9 and pL2Cas9) as determined by the double-layer plaque assay.

## Figures and Tables

**Table 1:** crRNA and tracrRNA nucleotide sequences.

RNA ID	Sequence (5′-3′)
crRNA1	AAUUGUUUUUCACUAGAUA*GUUUUAGAGCUGUGUUGUUUCGAA*
crRNA2	AUUUUCCUUUCUAUUAUCU*GUUUUAGAGCUGUGUUGUUUCGAA*
crRNA3	AUAUCAAGCUCCGCUUGCU*GUUUUAGAGCUGUGUUGUUUCGAA*
crRNA5	CCAUCAAAGACCCUAACCC*GUUUUAGAGCUGUGUUGUUU*
tracrRNA	UUGGAACUAUUCGAAACAACACAGCAAGUUAAAAUAAGGUUUAUCCGUAUUCAACUUGAAAAAGUGCGCACCGAUUCGGUGCUUUUUUA

Nucleotides in the crRNAs matching the CRISPR repeats are in italics.

**Table 2:** Titres of phage p2 on various *L. cremoris* strains and analysis of *orf49* in the resulting phage plaques.

Strains	Titre (PFUs/mL)	EOP	WT	Deletion Mutant	Mixed Population
pTRKL2	3.0 ± 0.5 × 10^8^	N/A	96	0	4
SmutCas9	3.4 ± 0.7 × 10^8^	N/A	94	0	6
SmutCas9-49	2.9 ± 0.1 × 10^8^	0.85	0	0	100
pL2Cas9	3.9 ± 0.7 × 10^8^	N/A	98	0	2
pL2Cas9-49	1.9 ± 0.1 × 10^7^	0.05	0	100	0

In the first column, the *L. cremoris* MG1363-pKO49 variants are listed and identified by the pTRKL2-based construct that was transformed into them. The titre of phage p2 on each of these strains is in the second column and the EOPs in the third column. N/A means Not Applicable. The presence of *orf49* in various phages, determined by PCR, is shown in the fourth, fifth, and sixth columns. The 100 tested phage plaques were classified as either wild-type (WT), deletion mutant, or mixed population.

**Table 3:** Titres of phage p2 on various *L. cremoris* strains and analysis of *orf44* in the resulting phage plaques.

Strains	Titre (PFUs/mL)	EOP	WT	Deletion Mutant	Mixed Population
pTRKL2	2.5 ± 0.1 × 10^8^	N/A	0	0	100
SmutCas9	2.6 ± 0.7 × 10^8^	N/A	0	0	100
SmutCas9-44	2.5 ± 0.1 × 10^8^	0.96	0	0	100
pL2Cas9	2.2 ± 1.3 × 10^8^	N/A	0	0	100
pL2Cas9-44	2.6 ± 0.1 × 10^4^	1.2 × 10^−4^	1	0	99

In the first column, the *L. cremoris* MG1363-pKO44 variants are listed identified by the pTRKL2-based construct that was transformed into them. Titres, EOPs, and the presence of *orf44* determined by PCRs are found in columns two to six.

**Table 4:** List of primers used.

Primer	Sequence (5′-3′)
tracrRNA_F	AACTATTCGAAACAACACAG
PolyT_R	TTTTTTTTTTTTTTTTTTTTTTTTTTTTTT
CB13.10	ACCTCCTGCAAAGTCATCTG
CB13.42	GCAAATGACAGAAGAACAGC
CB14.6	GCTAAAACCGAACAATAAATGTC
p2.27	GCACAACCTATTGTAAAACC
pTRKL2_F	AAATATAGAAATATTTCTGTATTTTTTGGGCCAGTGAATTCCCGGGGATC
pTRKL2_R	AAGTGTCTTTTATGGGATTTTCTTTAAATCTTCTATTTAATCACTTTGAC
SmutCas9_F	GTCAAAGTGATTAAATAGAAGATTTAAAGAAAATCCCATAAAA
SmutCas9_R	GATCCCCGGGAATTCACTGGCCCAAAAAATACAGAAATATTTC
spacer44_F	*AAAC*TAGCCATGTTTTTATCTCCTTTCTTGATGA*G*
spacer44_R	*AAAAC*TCATCAAGAAAGGAGATAAAAACATGGCTA
spacer49_F	*AAAC*CATCTATCTTATTGGTAGTGGCTGGAGTAT*G*
spacer49_R	*AAAAC*ATACTCCAGCCACTACCAATAAGATAGATG

Restriction sites for BsaI are in italics.

## Data Availability

Data is contained within the article and Supplementary Materials.

## Supplementary Materials

The following supporting information can be downloaded at: https://www.mdpi.com/article/10.3390/ijms26052005/s1.
