# Supplementary material for: Insight into crRNA Processing in Streptococcus mutans P42S and Application of SmutCas9 in Genome Editing
Source: Int J Mol Sci. 2025 Feb 25;26(5):2005. doi: 10.3390/ijms26052005 (PMC11900481; doi:10.3390/ijms26052005)
Supplement: Supplementary file 1 [file ijms-26-02005-s001.zip › ijms-3490491-supplementary.pdf]

Supplementary data

Table S1: Sequence pTRKL2-SmutCas9. SmutCas9 is in *italics*.

```
1       10        20        30        40        50
|       |         |         |         |         |
AAGGAGAACAGCTGAATGAATATCCCTTTTGTTGTAGAAACTGTGCTTCA
TGACGGCTTGTTAAAGTACAAATTTAAAAATAGTAAAATTCGCTCAATCA
CTACCAAGCCAGGTAAAAGCAAAGGGGCTATTTTTGCGTATCGCTCAAAA
TCAAGCATGATTGGCGGTCGTGGTGTTGTTCTGACTTCCGAGGAAGCGAT
TCAAGAAAATCAAGATACATTTACACATTGGACACCCAACGTTTATCGTT
ATGGAACGTATGCAGACGAAAACCGTTCATACACGAAAGGACATTCTGAA
AACAATTTAAGACAAATCAATACCTTCTTTATTGATTTTGATATTCACAC
GGCAAAAGAAACTATTTCAGCAAGCGATATTTTAACAACCGCTATTGATT
TAGGTTTTATGCCTACTATGATTATCAAATCTGATAAAGGTTATCAAGCA
TATTTTGTTTTAGAAACGCCAGTCTATGTGACTTCAAAATCAGAATTTAA
ATCTGTCAAAGCAGCCAAAATAATTTCGCAAAATATCCGAGAATATTTTG
GAAAGTCTTTGCCAGTTGATCTAACGTGTAATCATTTTGGTATTGCTCGC
ATACCAAGAACGGACAATGTAGAATTTTTTGATCCTAATTACCGTTATTC
TTTCAAAGAATGGCAAGATTGGTCTTTCAAACAAACAGATAATAAGGGCT
TTACTCGTTCAAGTCTAACGGTTTTAAGCGGTACAGAAGGCAAAAAACAA
GTAGATGAACCCTGGTTTAATCTCTTATTGCACGAAACGAAATTTTCAGG
AGAAAAGGGTTTAATAGGGCGTAATAACGTCATGTTTACCCTCTCTTTAG
CCTACTTTAGTTCAGGCTATTCAATCGAAACGTGCGAATATAATATGTTT
GAGTTTAATAATCGATTAGATCAACCCTTAGAAGAAAAAGAAGTAATCAA
AATTGTTAGAAGTGCCTATTCAGAAAACTATCAAGGGGCTAATAGGGAAT
ACATTACCATTCTTTGCAAAGCTTGGGTATCAAGTGATTTAACCAGTAAA
GATTTATTTGTCCGTCAAGGGTGGTTTAAATTCAAGAAAAAAAGAAGCGA
ACGTCAACGTGTTCATTTGTCAGAATGGAAAGAAGATTTAATGGCTTATA
TTAGCGAAAAAAGCGATGTATACAAGCCTTATTTAGTGACGACCAAAAAA
GAGATTAGAGAAGTGCTAGGCATTCCTGAACGGACATTAGATAAATTGCT
GAAGGTACTGAAGGCGAATCAGGAAATTTTCTTTAAGATTAAACCAGGAA
GAAATGGTGGCATTCAACTTGCTAGTGTTAAATCATTGTTGCTATCGATC
ATTAAAGTAAAAAAAGAAGAAAAAGAAAGCTATATAAAGGCGCTGACAAA
TTCTTTTGACTTAGAGCATACATTCATTCAAGAGACTTTAAACAAGCTAG
CAGAACGCCCTAAAACGGACACACAACTCGATTTGTTTAGCTATGATACA
GGCTGAAAATAAAACCCGCACTATGCCATTACATTTATATCTATGATACG
TGTTTGTTTTTTCTTTGCTGTTTAGCGAATGATTAGCAGAAATATACAGA
GTAAGATTTTAATTAATTATTAGGGGGAGAAGGAGAGAGTAGCCCGAAAA
CTTTTAGTTGGCTTGGACTGAACGAAGTGAGGGAAAGGCTACTAAAACGT
CGAGGGGCAGTGAGAGCGAAGCGAACACTTGATTTTTTAATTTTCTATCT
TTTATAGGTCATTAGAGTATACTTATTTGTCCTATAAACTATTTAGCAGC
ATAATAGATTTATTGAATAGGTCATTTAAGTTGAGCATATTAGAGGAGGA
AAATCTTGGAGAAATATTTGAAGAACCCGATTACATGGATTGGATTAGTT
CTTGTGGTTACGTGGTTTTTAACTAAAAGTAGTGAATTTTTGATTTTTGG
TGTGTGTGTCTTGTTGTTAGTATTTGCTAGTCAAAGTGATTAAATAGAAG
ATTTAAAGAAAATCCCATAAAAGACACTTGAATTATTGCTTCAAGTGTCT
TTTGCTTATTTAAAAATCTTTCGCATAATGTGTTTTAGTAAATCAGTAAC
TAACATGGCTCTAAAACATGTGCCGCCTTTACGGATTATATGTGCCAGTT
TTGGAACCATTCGAAACAACACAGCTCTAAAACGAGACCTTTGAGCTTCC
GAGACTGGTCTCAGTTTTGGAACCATTCGAAACAACACAGCTCTAAAACC
```

TCGTGGACCAATTTTCGTCACGACATTTCGTAATCGCGCCATTCATCTCA
GCTAGAATTCAGCTTCAAATAATTCCTTTTTAAAAGATTTTAATTAATTA
TATTATACCTAAAAAAGTGCTAGAAATCAATCTTTCTAGCGCTTTTCTTG
TTTTATAATTGTAGTTTCCTTTCCAAAGCTTTTGTTAATACTTCAGAATA
ATTCACTTGATCACGATCTGCTAATTGTACTAGATTAGTCTCCTCCTAAC
TTACTGAGATCAATCCGCGTTTCGTAAAGACCGGTGATGGATTGGTGGAT
GAGGGTAGCCTCCATACATGGTAAAAGATAATCTTTCTTACCTGTATATT
GTTTTTGATTAAGTTTTATCCCTAAAAAGCTAAATGGAGAGGTCGCTCCC
AATTGAGTAAATCCTAATAATTTAATAAAACCATCAGCTAATTCCTCAAT
TGAAGCAGAATCCCGCTCCTTCGAATAAGCTTCTTCAATTTTTTGAATGT
GCTCTTTCCCTAGTTTACATTTTTTAGAAAAAGAAGTAATTTCATTAAAC
AAAATATCAAATTGTTGATAATGATTTTTAACATATTCATTACTATAATC
GTTGTCTAAATGATGAGCATGATAAAGTAAATCTTGAGATTTTTTAGACA
CAACCAATTGATTTCCTTTATGTACTTCTTTTGAGCTAGCCCATAAACGT
TTAATGCCATTGCCAATATCTACTAGAGTGTATTTAGGTAATTTAACAAA
GTTATTCTTTTCTATCTGTTGATAACCTTTATCTTTCAAAAATTTAACAG
GATTTTGTTCAAATCGTTTCTTATCCATTACTGAAATTGGAATTAACTGT
TTTGTATCTACTATTAATAAAATTGGATAAGCTGTTGTTGGTTTTTGATA
GCCTCCATATTTCTCAGGATTTAATGCTTTTTTTAGTGGAACAAGCTTAC
TAGGTATAACCTCTAATGGCGATTTAGGATTATCATCAAAAAGCCCCCCA
TTTTGTCCAATGGTTTGTTCCTCCACTTTCTTAACAATATTGACTTGTGG
ATAAGAAAGAACTTTTTTGACAGTAGTGATATGCTTTGTTTTATCCCAAG
CAATTTCTCCTGTTTCATCATTTACTTCAATCATCGGACGTTCGACAATT
TGATCATCTGCCAATTTGACTTTGGACTTGAACATATTCATGATATTAGA
GTAGAAAAACATCTTTTGCGTTGCTGTTTTACGCTCACGATAGCTATTGT
ATTTTGGATATTCACCGTAAACAAATTCTGGTTCCAATTTTGGATATTTT
ACGAGAAGAGCTTTGACAACAACCGCGTTAAGATAAGCATCATGTGCGTG
GTGATAATCGTTAATTTCACGGACTTTATAAAGTTCAAATTCTTTTCGGA
AAGATGACACAAGATTTGATTTCAAAGTGACAATTTTGACCCTGCGAATA
CGTTTATTATTGTCATCAAATTCTTTATTAAAGCGTTCATCAAGCATACG
TGCCACGTGTTTAGTGATTTGTCTGGTTTCAACCAATTGGCGTTTAATAA
AGCCAGCCTTATCATCTGGTGTCAATTCTTTCTTGGTTAGATTGTTATAT
TTCCGTTGAGAAATCAAGCCACTACTCAACAACTTATTCCAAAACGGACG
CATCCTATTGACAACATCTTCACTTGGTACATCATCCGATTTTCCACGAT
TTTCCTTTGAGCTAGTCAATACTCTATTATCAATAGAATTATCCTTGATA
AAAGCTTGCGGGATAATATGGTCTATATCATACTGGCTTAGATAATCAAT
ATCCAATTCTTCTCCAGTATACATATCTCTGCCGTTTTGTAAATAATATA
GAAACAATCTATCATTTTGTAACTGTGAATTCTCAACCGGATGTTCTTTA
AGAATTTGACTTCCAAATTCTTTAATAGAATCTGTCAAACCTTTCAAACG
CTGCTGTGAATTTCGTCGTCCCTGATTGGTAAACTGGTTTTCACGCGCCA
TCTCCACGACGATATTTTCAGGTTGATGTCCCATAATTTTGACAAGCTCA
TCAACAATCTTCAAGCTTTGTAAAATTCCTTTTTTAATAGCAGGGCTGCC
AGCAATATCACTAACAACTTGATTTAGATTGTCTGTTTCTCCAATAACTT
GTGCCTTAGCAATCTCTTCTTTGAAAGAAAGAGCATCATCGTTAATCAGT
TGCATAAAGTTACGATTGCTATTGCCATCATCAATGAGATAATCAAGAAT
TGTTTTTCTGCTTTCTTTGTTGCGAATACCATGAATTAACTCAGCTGATA
ATCTTCCCCAACCAGTATAATGACGTCTTTCCAGCTTTTTCACTTGTTCT
TTGGTCAATAAATCACTGTAATTTTCTAGACGTTTTCTAATCATTTCTCT
ATCTTCAAATAACGTTAAGGTCAACACAATATCTTCTAAAATCTTTTCAT
TCTTTGAATTATCGAGAAAATCTTTATCTAAAATTTTACGCAAATCATGA
TAAGTTCCATAAGAAGCGTTAAATGCTTTATTTTCTTTATCCAGACCTGT

TAAATCAACAATACGAAATTCATCAAATTCTTTTTCAAGGAAATCCATTA
ATTTATCTTTAGTTACTTTTCGATAAACCTTAAATACGCCATCAAAGATT
TCTTGCTTCATATTGGCATCAAAAAATGCTGTTTTTCCTTGCTCTGTTTT
ATATTTAACCTTTGTTAATTCATTGTAAACAGTAAATTTTTCGTATAATA
AACTATGTTTAGGAAGAACTTTTTGATTTGGCAAGTACAAATCATAATTT
GTCATACGATTGATAAAAGCTTCTGCAGAGGATTCTTTATCAACGATTTC
ATCAAAATTCCATGGTGTAATTTTATCAGCCGATTTCCGACTTAACCAAG
CAAAATCACTTTTTCCGCGCGCTAATGGACCAACATAGTAGGGAATACGG
AAAGTCAATATTTTCTCAATCCTATCTTGATTGTCTGCTAAAAACGGATA
AAATTCAGCCTGTCTACGAATGATAGCACGCATTTCTTGAAGATGAATCT
GATGTGGAATAGAGCCATTGTCAAAGGTACGTTGCTTTCTTAGAAAATCT
TCACGCTCAATTTTATCAAGGAAATAGCCACTTCCCTCAATCTTATTTAA
TAGACCTTTAAGGTATTTATAAAAAGCTTCTTGATTTGTTTTCCCATCAA
TATAACCCGCATAGCCGTCTTTTGAAACATCAGAAAAAACTTCGTTATAT
TTATCTGATAATTTCTGACGAATGAATTGTTTAAGCTGAGCTAAATCCAT
CTGATGTTCATTATATCGCTGAATCATCGAAGCAGATAAAGGCGCTTTGG
TACTAACATCAGTAACTGTTAAAATCCCTGATAAAAGGATACTATCATAC
AGTTTCTTTGCTGATAAAAAGAGCTCTGCGTAATTATCTCCAATTTGAGC
TAATAGTACTTCTAACTCTTCTTCATAAGTATCTTTAGAAAATTGCAATG
GTGTTTTCTCTTCTAATTCAAAATGCTTTTTAAAATCAGCTTGATTACCA
ACAATTAGTTTTAGAAATTCTGCAAAGCGGCCATTAGACTTTTCATTAGG
AAAAAGTTTCAAAACTCTATCTTTCTTAGCAGACTTACTGATTTTATCAG
TCAGAATTTCCTCAACTTGAACATTTTGCTCCTGAAGCGAACTATTCTCA
AAAGTATTATCATAGACTGCTAAAAATTCTTGAAACAGTCTTTGTACATC
ATTATTGCGTGTATCAAACTTTCCTTCAATTAAAAAATGGCCTCTAAACT
TAATTATATGTGCCAAAGCCAAATAAACTAAACGCAAATCAACCTTTTCT
GGATTATCCGCAAGATATTGCCGCAAATGATAAATGGTTGGAAAATTTTC
ATGATACTTAACTTCTTCTTCAAGATTCCCAAAAATGGGATGGCGCTCTC
CTCGTTTATCCTCAGTAACAAGAAAAGAATCCTCTAAACGATGAAAGAAA
CTATCATCTACCTTGCTCATTTCTTCTGCAAAAATCTCTTGCAAATATAA
AATACGATTTCTACGACGTGTATAACGACGGCGAGCTGTACGCTTTAAAC
GTCTATCTGCTGCAGTATTCCCGCTATCAAATAATAAAGCGCCAAGCAAA
TTTTTCTTGATATGACTTTTATCTGTATTTCCCAGAACCTTCATCTTCTT
AGCAGGAACCTTGTAGTCATCTGTCACAACAGCCCAACCAACAGAATTGG
TTCCAATATCAAGTCCAATAGAGTAAGGTTTTTTCATAAATGTCTCCTTT
GAAAAAATGTCCTTGCAAAAAAGCAATAAGTATTATACAATAATTGTGTT
GGAACTATTCGAAACAACACAGCAAGTTAAAATAAGGTTTATCCGTATTC
AACTTGAAAAAGTGCGCACCGATTCGGTGCTTTTTTATTTGCTTTACTGT
AAGCGTTTCTATTATACCACTATCTACGAAATATTTTAACAATATAAACG
*AAAAAATATAGAAATATTTCTGTATTTTTTGGG*CCAGTGAATTCCCGGGG
ATCCGTCGACCTGCAGCCAAGCTTTCGCGAGCTCGAGATCTAGATATCGA
TGAATTCGTAATCATGGTCATAGCTGTTTCCTGTGTGAAATTGTTATCCG
CTCACAATTCCACACAACATACGAGCCGGAAGCATAAAGTGTAAAGCCTG
GGGTGCCTAATGAGTGAGCTAACTCACATTAATTGCGTTGCGCTCACTGC
CCGCTTTCCAGTCGGGAAACCTGTCGTGCCAGCTGCATTAATGAATCGGC
CAACGCGCGGGGAGAGGCGGTTTGCGTATTGGAATTCCGGATGAGCATTC
ATCAGGCGGGCAAGAATGTGAATAAAGGCCGGATAAAACTTGTGCTTATT
TTTCTTTACGGTCTTTAAAAAGGCCGTAATATCCAGCTGAACGGTCTGGT
TATAGGTACATTGAGCAACTGACTGAAATGCCTCAAAATGTTCTTTACGA
TGCCATTGGGATATATCAACGGTGGTATATCCAGTGATTTTTTTCTCCAT
TTTAGCTTCCTTAGCTCCTGAAAATCTCGATAACTCAAAAAATACGCCCG

GTAGTGATCTTATTTCATTATGGTGAAAGTTGGAACCTCTTACGTGCCGA
TCAACGTCTCATTTTCGCCAAAAGTTGGCCCAGGGCTTCCCGGTATCAAC
AGGGACACCAGGATTTATTTATTCTGCGAAGTGATCTTCCGTCACAGGTA
TTTATTCGGCGCAAAGTGCGTCGGGTGATGCTGCCAACTTACTGATTTAG
TGTATGATGGTGTTTTTGAGGTGCTCCAGTGGCTTCTGTTTCTATCAGCT
GTCCCTCCTGTTCAGCTACTGACGGGGTGGTGCGTAACGGCAAAAGCACC
GCCGGACATCAGCGCTAGCGGAGTGTATACTGGCTTACTATGTTGGCACT
GATGAGGGTGTCAGTGAAGTGCTTCATGTGGCAGGAGAAAAAAGGCTGCA
CCGGTGCGTCAGCAGAATATGTGATACAGGATATATTCCGCTTCCTCGCT
CACTGACTCGCTACGCTCGGTCGTTCGACTGCGGCGAGCGGAAATGGCTT
ACGAACGGGGCGGAGATTTCCTGGAAGATGCCAGGAAGATACTTAACAGG
GAAGTGAGAGGGCCGCGGCAAAGCCGTTTTTCCATAGGCTCCGCCCCCCT
GACAAGCATCACGAAATCTGACGCTCAAATCAGTGGTGGCGAAACCCGAC
AGGACTATAAAGATACCAGGCGTTTCCCCCTGGCGGCTCCCTCGTGCGCT
CTCCTGTTCCTGCCTTTCGGTTTACCGGTGTCATTCCGCTGTTATGGCCG
CGTTTGTCTCATTCCACGCCTGACACTCAGTTCCGGGTAGGCAGTTCGCT
CCAAGCTGGACTGTATGCACGAACCCCCCGTTCAGTCCGACCGCTGCGCC
TTATCCGGTAACTATCGTCTTGAGTCCAACCCGGAAAGACATGCAAAAGC
ACCACTGGCAGCAGCCACTGGTAATTGATTTAGAGGAGTTAGTCTTGAAG
TCATGCGCCGGTTAAGGCTAAACTGAAAGGACAAGTTTTGGTGACTGCGC
TCCTCCAAGCCAGTTACCTCGGTTCAAAGAGTTGGTAGCTCAGAGAACCT
TCGAAAAACCGCCCTGCAAGGCGGTTTTTTCGTTTTCAGAGCAAGAGATT
ACGCGCAGACCAAAACGATCTCAAGAAGATCATCTTATTAATCAGATAAA
ATATTTCTAGACTCGAGAGCTCTCGAGTCTAGAATCGATACGATTTTGAA
GTGGCAACAGATAAAAAAAAGCAGTTTAAAATTGTTGCTGAACTTTTAAA
ACAAGCAAATACAATCATTGTCGCAACAGATAGCGACAGAGAAGGCGAAA
ACATTGCCTGGTCGATCATTCATAAAGCAAATGCCTTTTCTAAAGATAAA
ACGTATAAAAGACTATGGATCAATAGTTTAGAAAAAGATGTGATCCGTAG
CGGTTTTCAAAATTTGCAACCAGGAATGAATTACTATCCCTTTTATCAAG
AAGCGCACAAAAAGAAAAACGAAATGATACACCAATCAGTGCAAAAAAAG
ATATAATGGGAGATAAGACGGTTCGTGTTCGTGCTGACTTGCACCATATC
ATAAAAATCGAAACAGCAAAGAATGGCGGAAACGTAAAAGAAGTTATGGA
AATAAGACTTAGAAGCAAACTTAAGAGTGTGTTGATAGTGCAGTATCTTA
AAATTTTGTATAATAGGAATTGAAGTTAAATTAGATGCTAAAAATTTGTA
ATTAAGAAGGAGTGATTACATGAACAAAAATATAAAATATTCTCAAAACT
TTTTAACGAGTGAAAAAGTACTCAACCAAATAATAAAACAATTGAATTTA
AAAGAAACCGATACCGTTTACGAAATTGGAACAGGTAAAGGGCATTTAAC
GACGAAACTGGCTAAAATAAGTAAACAGGTAACGTCTATTGAATTAGACA
GTCATCTATTCAACTTATCGTCAGAAAAATTAAAACTGAATACTCGTGTC
ACTTTAATTCACCAAGATATTCTACAGTTTCAATTCCCTAACAAACAGAG
GTATAAAATTGTTGGGAGTATTCCTTACCATTTAAGCACACAAATTATTA
AAAAAGTGGTTTTTGAAAGCCATGCGTCTGACATCTATCTGATTGTTGAA
GAAGGATTCTACAAGCGTACCTTGGATATTCACCGAACACTAGGGTTGCT
CTTGCACACTCAAGTCTCGATTCAGCAATTGCTTAAGCTGCCAGCGGAAT
GCTTTCATCCTAAACCAAAAGTAAACAGTGTCTTAATAAAACTTACCCGC
CATACCACAGATGTTCCAGATAAATATTGGAAGCTATATACGTACTTTGT
TTCAAAATGGGTCAATCGAGAATATCGTCAACTGTTTACTAAAAATCAGT
TTCATCAAGCAATGAAACACGCCAAAGTAAACAATTTAAGTACCGTTACT
TATGAGCAAGTATTGTCTATTTTTAATAGTTATCTATTATTTAACGGGAG
GAAATAATTCTATGAGTCGCTTTTGTAAATTTGGAAAGTTACACGTTACT
AAAGGGAATGTAGATAAATTATTAGGTATACTACTGACAGCTTCCAAGGA

GCTAAAGAGGTCCCTAGCGCTTAGAATCGCTTTAGGAAACACGATCCAGT
CCAATAATCGTCGATAAAAACTTTTGAAAAAGGTTGGTGAAATTACCTAC
TTTTGGAATAATCACAAATCACAAGTGATTAATCACAAATCACAAGTGAT
TAATCACTTGTTTATTAAGATATTAAAAGCTATAATTTAAATAAAGCGTG
AATTTTATTACACAAAAAGAGGGGGGAGAAACTTGGAACTAGCATTTAGA
GAAAGCTTAAAAAAGATGAGAGGTACCAAATCAAAAGAAAAATTCTCCCA
AGAATTAGAAATGAGTAGATCAAATTATTCACGAATAGAATCAGGAAAAT
CAGATCCAACCATAAAAACACTAGAACAAATTGCAAAGTTAACTAACTCA
ACGCTAGTAGTGGATTTAATCCCAAATGAGCCAACAGAACCAGAACCAGA
AACAGAATCAGAACAAGTAACATTGGATTTAGAAATGGAAGAAGAAAAAA
GCAATGACTTCGTGTGAATAATGCACGAAATCGTTGCTTATTTTTTTTAA
AAGCGGTATACTAGATATAACGAAACAACGAACTGAATAGAAACGAAAAA
AGAGCCATGACACATTTATAAAATGTTTGACGACATTTTATAAATGCATA
GCCCGATAAGATTGCCAAACCAACGCTTATCAGTTAGTCAGATGAACTCT
TCCCTCGTAAGAAGTTATTTAATTAACTTTGTTTGAAGACGGTATATAAC
CGTACTATCATTATATAGGGAAATCAGAGAGTTTTCAAGTATCTAAGCTA
CTGAATTTAAGAATTGTTAAGCAATCAATCGGAAATCGTTTGATTGCTTT
TTTTGTATTCATTTATAGAAGGTGGAGTTTGTATGAATCATGATGAATGT
AAAACTTATATAAAAAATAGTTTATTGGAGATAAGAAAATTAGCAAATAT
CTATACACTAGAAACGTTTAAGAAAGAGTTAGAAAAGAGAAATATCTACT
TAGAAACAAAATCAGATAAGTATTTTTCTTCGGAGGGGGAAGATTATATA
TATAAGTTAATAGAAAATAACAAAATAATTTATTCGATTAGTGGAAAAAA
ATTGACTTATAAAGGAAAAAAATCTTTTTCAAAACATGCAATATTGAAAC
AGTTGAATGAAAAAGCAAACCAAGTTAATTAAACAACCTATTTTATAGGA
TTTATAGGAA
